# Differential effects of reproductive factors on the risk of pre- and postmenopausal breast cancer. Results from a large cohort of French women

**DOI:** 10.1038/sj.bjc.6600124

**Published:** 2002-03-04

**Authors:** F Clavel-Chapelon, and the E3N-EPIC Group

**Affiliations:** Equipe E3N-EPIC, INSERM U521, Institut Gustave-Roussy, 94805 Villejuif cedex, France

**Keywords:** breast cancer, cohort study, risk factors, reproductive factors

## Abstract

The aim of this study was to obtain a better understanding of the role of hormonal factors in breast cancer risk and to determine whether the effect of reproductive events differs according to age at diagnosis. It analysed the effect of age at menarche, age at first full-term pregnancy, number of full-term pregnancies and number of spontaneous abortions both on the overall risk of breast cancer and on its pre- or postmenopausal onset, using the data on 1718 breast cancer cases, obtained from a large sample of around 100 000 French women participating in the E3N cohort study. The results provide further evidence that the overall risk of breast cancer increases with decreasing age at menarche, increasing age at first pregnancy and low parity. No overall effect of spontaneous abortions was observed. The effect of these reproductive factors differed according to menopausal status. Age at menarche had an effect on premenopausal breast cancer risk, with a decrease in risk with increasing age of 7% per year (*P*<0.05). Compared to those who had their first menstrual periods at 11 or before, women experiencing menarche at 15 or after had an RR of 0.66 (95% CI 0.45–0.97) in the premenopausal group. Age at first full-term pregnancy had an effect on both pre- and postmenopausal breast cancer risk, with significant tests showing increasing risk per year of increasing age (*P*=0.001 and *P*<0.05 respectively). A first full-term pregnancy above age 30 conveyed a risk of 1.63 (95% CI 1.12–2.38) and 1.35 (95% CI 1.02–1.78) in the pre- and postmenopausal groups respectively. A protective effect of high parity was observed only for postmenopausal breast cancer risk (*P* for trend test =0.001), with point estimates of 0.79 (95% CI 0.60–1.04), 0.69 (95% CI 0.54–0.88), 0.66 (95% CI 0.51–0.85) and 0.64 (95% CI 0.48–0.86) associated to a one, two, three and four or more full-term pregnancies. A history of spontaneous abortion had no significant effect on the risk of breast cancer diagnosed before or after menopause. Our results suggest that reproductive events have complex effects on the risk of breast cancer.

*British Journal of Cancer* (2002) **86**, 723–727. DOI: 10.1038/sj/bjc/6600124 www.bjcancer.com

© 2002 Cancer Research UK

## 

There is a considerable body of evidence that reproductive factors play a role in the aetiology of breast cancer risk (Kelsey *et al*, 1993). However, the biological mechanism is not yet clearly understood. Results showing the effect of these factors by age or menopausal status at the time of diagnosis may help to give a better understanding of their role.

A pooled analysis of the literature showed that early age at menarche and late age at first full-term pregnancy (FFTP) had a greater effect on the risk of breast cancers diagnosed early (i.e. at an early age or before menopause) than on the risk of those diagnosed late (i.e. after 55 or after menopause). In contrast, it has been shown that the protective effect of multiparity is greater on late breast cancers (Clavel-Chapelon and Gerber, 2001). Epidemiological studies reporting on the relationship between breast cancer risk and spontaneous or induced abortions by age have produced conflicting results (Michels and Willett, 1996; Wingo *et al*, 1997).

We report here on the effects of age at menarche, age at FFTP, number of full-term pregnancies (FTP) and number of spontaneous abortions both on the overall risk of breast cancer and on its pre- or postmenopausal onset, using data obtained from a large sample of French women participating in the E3N cohort study.

## MATERIALS AND METHODS

E3N is a prospective cohort study on cancer risk factors, conducted in France (Clavel-Chapelon, 1997). Part of the E3N cohort, i.e. women who replied to the dietary questionnaire, are included in the European Prospective Investigation on Cancer (EPIC) (Riboli, 1992).

The cohort consists of 100 000 women living in France who are insured with the Mutuelle Générale de l'Education Nationale, a national health insurance scheme primarily covering teachers. They were aged 40–65 at baseline. The main objective of the study was to investigate risk factors for cancer and other diseases (cardiovascular diseases, diabetes and osteoporosis). Participants were enrolled in the study between June 1990 and November 1991 after replying to a baseline questionnaire containing questions on lifestyle, i.e. reproductive factors, body build, smoking, past medical history and family history of cancer. Follow-up questionnaires were sent out at approximately 24-month intervals until April 1997.

Age at menarche, age at FFTP, overall number of spontaneous abortions and number of FTP were recorded in the two first questionnaires. Menopausal status was recorded in each questionnaire. Postmenopause was defined as the cessation of periods for natural reasons or due to surgery (total oophorectomy). Women with undefined menopausal status (for instance due to continuous use of hormonal treatments, or hysterectomy with no additional information on oophorectomy) were considered in the premenopausal group up to 42 years of age, and in the postmenopausal one after 58. They were not considered in the analysis by subgroup between these limits (i.e. (42–58) years of age, defined as the mean age at menopause in our population ±2 s.d.), as were women who never menstruated.

All questionnaires asked participants whether breast cancer had been diagnosed, requesting the addresses of their physicians and permission to contact them. Deaths in the cohort were detected from reports by family members or by the postal service and by a search on the insurance company (MGEN) file, which contains information on vital status. Information on the cause of death was obtained from the National Service on Causes of Deaths (INSERM). Information on non-respondents was obtained from the MGEN file on reimbursement of hospital fees. In this case, the subject's physician was contacted for diagnostic information. The procedure made it possible to find additional breast cancer cases (*n*=39). Only 1815 women could not be traced in the MGEN file (names misspelled, names changed after divorce, no longer insured with the MGEN, etc.). Among these, non respondents were considered lost to follow-up.

Follow-up time was between return of the baseline questionnaire and December 1997, the date by which most copies (92%) of the fifth questionnaire (sent out in April 1997) had been returned. Participants contributed person-time up to the date of any diagnosis of breast cancer, date of death, date of last questionnaire returned or December 1997 (for replies to the April 1997 questionnaire received after December 1997), whichever occurred first. Women whose menopause occurred during the period of follow-up contributed person-time in the premenopausal group until the date of menopause and in the postmenopausal group thereafter.

A total of 2100 breast cancer cases were reported by participants. Pathology reports were obtained for 97% (*n*=2044) of cases. Of these, 94.5% confirmed the self-reported diagnosis of breast cancer. After exclusion of the 112 cases whose diagnosis was rejected as a result of the pathology report and 270 cases of carcinoma *in situ*, 1718 cases of invasive breast cancer were available for analysis, including the 56 cases whose diagnosis was based on the self-report only, as self-reporting proved to be extremely accurate. Histologic types were coded by a pathologist blinded to data on risk factors. Women who had reported a cancer other than a basal cell carcinoma at enrolment were excluded.

Statistical analyses were performed using SAS statistical software. A proportional hazard model with follow-up time as the time axis was used (Cox, 1972) allowing estimation of relative risks (RRs) and 95% confidence intervals. All reproductive factors were included simultaneously in the model to adjust for mutual confounding, since the four reproductive factors studied may influence each other. We also took into account as potential confounders age, personal history of benign breast disease, familial history of breast cancer, body mass index, marital status and educational level, because of their possible association with characteristics of the reproductive life and with breast cancer. Analyses were performed on the whole cohort, and further stratified by menopausal status.

Indeed, the whole population was split into three categories: premenopausal, postmenopausal and of unknown menopausal status. However, the vast majority of breast cancer cases were able to date their menopause from the date of radio- or chemotherapy. As a consequence, in that third category, the incidence rate was low and results are not presented here.

## RESULTS

Table 1

**Table 1:**
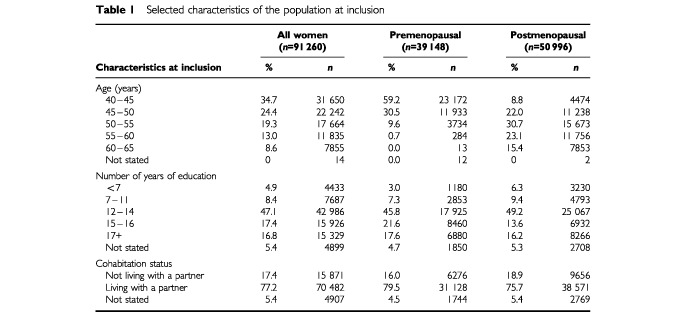
Selected characteristics of the population at inclusion

shows the distribution of all survey participants by age, education, cohabitation and menopausal status at baseline, and in separate strata of menopausal status. Among the 91 260 participants, 39 148 were premenopausal and 50 996 postmenopausal at the end of follow-up; menopause occurred during the follow-up for 16 026; for 17 138, menopausal status could not be defined; four never menstruated. A total of 1718 cases of invasive breast cancer were diagnosed in the 579 525 person–years of follow-up. Four hundred and seventy-eight cases occurred before menopause, 1057 after and 183 with undefined menopausal status were not considered in the analysis by subgroup.

Menstrual and reproductive factors are considered in Table 2

**Table 2:**
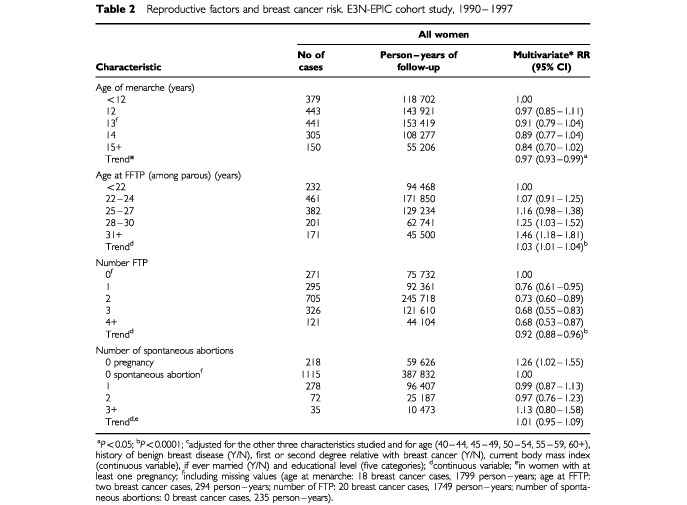
Reproductive factors and breast cancer risk. E3N-EPIC cohort study, 1990–1997

. A 3% decrease in breast cancer risk was found for each additional year at menarche (RR=0.97, 95% CI 0.93–0.99; *P*<0.05). Breast cancer risk increased with increasing age at FFTP by 3% per year (RR=1.03, 95% CI 1.01–1.04; *P*<10^−4^, among the 1447 parous women). Each FTP reduced the risk by 8% (RR=0.92, 95% CI 0.88–0.96; *P*<10^−4^). All estimates of the relationship between breast cancer risk and spontaneous abortions were non-significant.

Table 3

**Table 3:**
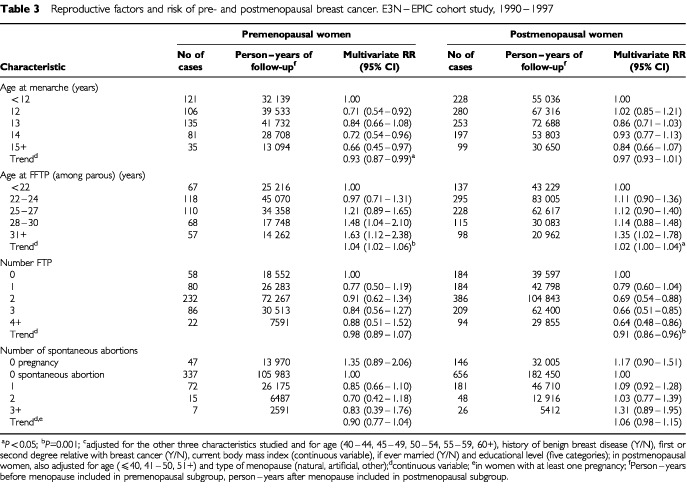
Reproductive factors and risk of pre- and postmenopausal breast cancer. E3N–EPIC cohort study, 1990–1997

shows the results according to menopausal status. The effect of age at menarche was similar in both subgroups. For each additional year in age at menarche, breast cancer risk decreased by 7% (RR=0.93, 95% CI 0.87–0.99; *P*<0.05) for breast cancers diagnosed before menopause and by 3% (RR=0.97, 95% CI 0.93–1.01) for those diagnosed later. An increase in age at FFTP increased the risk by 4% (RR=1.04, 95% CI 1.02–1.06; *P*=0.001) per year for breast cancer diagnosed in premenopausal women and by 2% (RR=1.02, 95% CI 1.00–1.04; *P*<0.05) per year for breast cancer diagnosed in postmenopausal women. The protective effect of an increasing number of FTPs was apparent only among postmenopausal women (RR=0.91, 95% CI 0.86–0.96; *P*<0.001). A history of spontaneous abortion had no significant effect on the risk of breast cancer diagnosed before or after menopause.

## DISCUSSION

The results provide further evidence that the overall risk of breast cancer increases with decreasing age at menarche, increasing age at first pregnancy and low parity. No overall effect of spontaneous abortions was observed. However, the effect of these reproductive factors differed according to menopausal status. Age at menarche had a significant effect on premenopausal breast cancer risk. Age at FFTP had a significant effect on the risk in both subgroups, the effect being slightly greater before menopause. Parity had a significant protective effect in the postmenopausal subgroup. A history of spontaneous abortions had no effect on the risk.

Consideration must be given to the validity of the data on reproductive factors. In a substudy of 751 women in the E3N study who completed the same self-administered questionnaire twice, we found a high rate of reproducibility for answers on age at menarche, age at first pregnancy, number of live births and duration of first pregnancy, with 71, 64, 98 and 80% respectively of identical answers. Nevertheless, even if assessment problems cannot be excluded, there should be no difference between cases and non-cases, given the prospective nature of the study. The reliability of the menopausal status and of age at menopause was assessed in a validation study (Clavel-Chapelon and Dormoy-Mortier, 1998). Agreement between the answer given in the initial questionnaire and the information collected from medical records of 151 women revealed a high concordance between the two sources (Kappa coefficient=0.85). Moreover, in the present study, women were categorised according to their menopausal status, after taking into account simultaneously, to correct for possible incoherences, all answers to the question on menopause, asked in the initial and all subsequent questionnaires.

Indeed, our results are remarkably close to those from the literature (Clavel-Chapelon and Gerber, 2001), though both chance and a lack of power may explain them.

On 21 studies, we showed that breast cancer risk decreased by about 9% (95% CI 7–11%) for each additional year in age at menarche for breast cancers diagnosed early or before menopause and by about 4% (95% CI 2–5%) for those diagnosed later. The hormonal levels to which girls are exposed increase continuously with age until menarche (Forrest and Bertrand, 1997) and it has been hypothesised that early menarche induces an early proliferation of breast cells through early exposure to high hormonal levels. However, a decrease in age at menarche has been described in many countries throughout the world. In our cohort, age at menarche decreased by 6 months over a period of 20 years, from 13.3 to 12.7 on average for the 1930 and 1950 birth cohorts respectively. This evolution is possibly due to several factors; for instance changes in food intake or in physical activity, which induce other hormonal changes in young girls, and may subsequently affect the breast cancer risk (Stoll, 1998).

In our previous analysis of 18 studies on age at FFTP, we found that each additional year led to an increased risk of 1.05 for early breast cancer and 1.03 for late breast cancer. According to the proposed multi-step process of carcinogenesis (Ponten *et al*, 1990), i.e. initiation, promotion, tumour and progression, undifferentiated cells, which have not undergone the maturation process, may be initiated by carcinogens and after promotion give rise to a breast tumour several years later. The differentiation of breast cells that occurs during the third trimester of pregnancy makes them less sensitive, or insensitive, to initiating agents. In other words the earlier the FFTP, the earlier the cells undergo differentiation. The proliferation of breast cells, which is greatest between 10 and 20 years of age, facilitates the promotion of the carcinogenic process.

Our previous review concluded that the relationship between breast cancer and parity, which had been investigated in 20 studies, resulted in a 3% (95% CI 1–6%) reduction in the risk of breast cancer diagnosed early or before menopause for each FTP, whereas the reduction rose to 12% (95% CI 10–14%) for breast cancers diagnosed later. The protective effect of multiparity is thus greater for cancers emerging late in life, and this agrees with recent studies that have found a transient increase in breast cancer risk immediately after each pregnancy (Lambe *et al*, 1994; Leon *et al*, 1995; Chie *et al*, 2000). The effect of multiparity might therefore derive from a short-term increase in risk followed by a long-term protective effect against late cancers. It could also be argued that the protective effect of multiparity may not be observed among young women simply because they are not usually multiparous.

Recent reviews on the relationship between breast cancer and spontaneous abortion (Michels and Willett, 1996; Wingo *et al*, 1997) did not come to any definitive conclusions, irrespective of whether study participants were under or over 45 years of age (Wingo *et al*, 1997).

Spontaneous abortions result in the lack of differentiation of the breast cells that occurs at the end of FTP. Spontaneous abortions that result from abnormally low production of progesterone by the corpus luteum may affect breast cancer risk as a consequence of low progesterone production. Whether the indication of a transient protection observed in our study on early breast cancer risk is due to the abrogation of the transient increase attributed to the final months of pregnancy, or is linked to the hormonal milieu, or is merely attributable to chance requires further investigation.
